# Genomic analysis reveals the association of *KIT* and *MITF* variants with the white spotting in swamp buffaloes

**DOI:** 10.1186/s12864-024-10634-2

**Published:** 2024-07-24

**Authors:** Dongmei Dai, Eka Meutia Sari, Jingfang Si, Hidayat Ashari, Muhammad Ihsan Andi Dagong, Alfredo Pauciullo, Johannes A. Lenstra, Jianlin Han, Yi Zhang

**Affiliations:** 1 State Key Laboratory of Animal Biotech Breeding, National Engineering Laboratory for Animal Breeding, Key Laboratory of Animal Genetics, Breeding and Reproduction of Ministry of Agriculture and Rural Affairs, College of Animal Science and Technology, China Agricultural University, Beijing, 100193 China; 2 Department of Animal Science, Agriculture Faculty, Universitas Syiah Kuala (USK), Banda Aceh, 23111 Indonesia; 3 Research Center for Biosystematics and Evolution, National Research and Innovation Agency (BRIN), Cibinong, 16911 Indonesia; 4 Animal Production Department, Faculty of Animal Science, Hasanuddin University, Makassar, 90245 Indonesia; 5 Department of Agricultural, Forest and Food Sciences, University of Turin, Grugliasco (TO), 10095 Italy; 6 Faculty of Veterinary Medicine, Utrecht University, Yalelaan 104, 3584 CM, Utrecht, The Netherlands; 7 Yazhouwan National Laboratory, Sanya, 572024 China

**Keywords:** White spotting, Swamp buffalo, *MITF*, *KIT*, Whole-genome sequencing

## Abstract

**Background:**

Swamp-type buffaloes with varying degrees of white spotting are found exclusively in Tana Toraja, South Sulawesi, Indonesia, where spotted buffalo bulls are highly valued in accordance with the Torajan customs. The white spotting depigmentation is caused by the absence of melanocytes. However, the genetic variants that cause this phenotype have not been fully characterized. The objective of this study was to identify the genomic regions and variants responsible for this unique coat-color pattern.

**Results:**

Genome-wide association study (GWAS) and selection signature analysis identified *MITF* as a key gene based on the whole-genome sequencing data of 28 solid and 39 spotted buffaloes, while *KIT* was also found to be involved in the development of this phenotype by a candidate gene approach. Alternative candidate mutations included, in addition to the previously reported nonsense mutation c.649 C > T (p.Arg217*) and splice donor mutation c.1179 + 2T > A in *MITF*, a nonsense mutation c.2028T > A (p.Tyr676*) in *KIT*. All these three mutations were located in the genomic regions that were highly conserved exclusively in Indonesian swamp buffaloes and they accounted largely (95%) for the manifestation of white spotting. Last but not the least, *ADAMTS20* and *TWIST2* may also contribute to the diversification of this coat-color pattern.

**Conclusions:**

The alternative mutations identified in this study affect, at least partially and independently, the development of melanocytes. The presence and persistence of such mutations may be explained by significant financial and social value of spotted buffaloes used in historical *Rambu Solo* ceremony in Tana Toraja, Indonesia. Several *de novo* spontaneous mutations have therefore been favored by traditional breeding for the spotted buffaloes.

**Supplementary Information:**

The online version contains supplementary material available at 10.1186/s12864-024-10634-2.

## Background

Coat color is one of the most visible phenotypic traits in animals and is under continuous selection in most domesticated animal species. It is largely determined by melanocytes, which produce melanins [1, 2]. After originating in the neural crest, melanoblasts migrate to various destinations, including the iris, dermis, and epidermis, where they differentiate into melanocytes determining the color of skin [2, 3]. The development of melanoblasts relies on the regulation of numerous transcription factors and signaling pathways, including the transcription factors PAX3, SOX10, LEF1 and MITF, G protein-coupled endothelin receptor B (EDNRB) and its ligand endothelin 3 (EDN3), and receptor tyrosine kinase KIT and KIT-ligand (KITLG) [3, 4]. However, disruption in the survival, migration, proliferation, and differentiation of melanoblasts may result in the absence of mature melanocytes, leading to white spotting [3, 5]. Due to the interaction of multiple genes that regulate the development of functional melanocytes, this phenotype was considered as a complex trait with patterns ranging from partially to completely white [6, 7]. A number of genes including *MITF*,* KIT*, *PAX3*, *EDN3*, *EDNRB*,* SNAI2*,* TRPM1*,* ADAMTS20* and *TWIST2* have been identified as candidate genes associated with white spotting in cattle [8–10], horses [11, 12], sheep [13, 14], and mice [15–18].

The swamp buffalo (*Bubalus bubalis carabanensis*) in Tana Toraja, South Sulawesi, Indonesia exhibit three coat color phenotypes, including solid black (wild type), spotted, and pure white coats (Figure S1) [19]. In the Toraja classification system, there are four types of spotted patterns, Saleko, Lotong Boko, Bonga, and Toddi [19]. Spotted buffalo bulls are sacrificed in funeral ceremonies and are valued ten times the price of solid black buffaloes [19–21]. The Toraja peoples’ perception and classification of buffaloes underlie the *Rambu Solo* ceremony, which is a traditional ritual for parents to become ancestors and reside in *Puya*, a life after death. For the Toraja people, this sacred ceremony is also related to prestige, dignity, and social status [22–26], which depend on the number and type of buffaloes that are sacrificed [22, 24–27]. The Toraja people search for buffaloes with a unique coat color pattern, large body size, and long and widespread horns [27]. After the slaughter, the distribution of meat while showing the buffalo heads highlight the social status of the owners [22, 24–27]. Therefore, the color pattern is under strong culture-driven selection in Indonesian swamp buffaloes [19].

A previous candidate gene study on Indonesian swamp buffaloes has identified two independent loss-of-function mutations in the *MITF* gene associated with white spotting [19]. However, these mutations cannot explain all the white-spotted phenotypes [19], implicating other mutations affecting the white spotting in swamp buffaloes.

In this study, we performed whole-genome sequencing (WGS) and applied a genome-wide association study (GWAS), combined with a candidate gene approach, to identify novel genetic variants associated with the white spotting in swamp buffaloes.

## Results

### *MITF* as a potential and functional candidate gene for the white spotting

Totally, 13,467,488 single nucleotide polymorphisms (SNPs) were retained for GWAS after quality control to identify the genes responsible for white spotting in swamp buffaloes (Figure S2A). We detected 208 genome-wide significant SNPs and 25 annotated genes associated with the white spotting (Table S1). Furthermore, a linkage disequilibrium-based cross-population extended haplotype homozygosity (XP-EHH) detection of selection signatures identified 853 annotated genes with the top 5% value as threshold (Figure S3). Enrichment analyses of the significant genes identified by XP-EHH indicated 232 gene ontology (GO) functional terms, including positive regulation of Wnt signaling pathway and pigmentation (Table S2). Notably, nine genes including *MITF*, *TBC1D4*, *LOC102412347*, *LOC102400532*, *LOC102399551*, *AURKC*, *ZNF805*, *TMEM132C*, and *OSBPL10* were identified both by GWAS and XP-EHH analysis. Among these genes, *MITF* was the only functional gene regulating the development of melanocytes and the transcription of melanogenic enzyme genes (Figure S2B, C) [3, 28].

### Nonsense and splice donor mutations in *MITF* associated with the white spotting

**Fig. 1:**
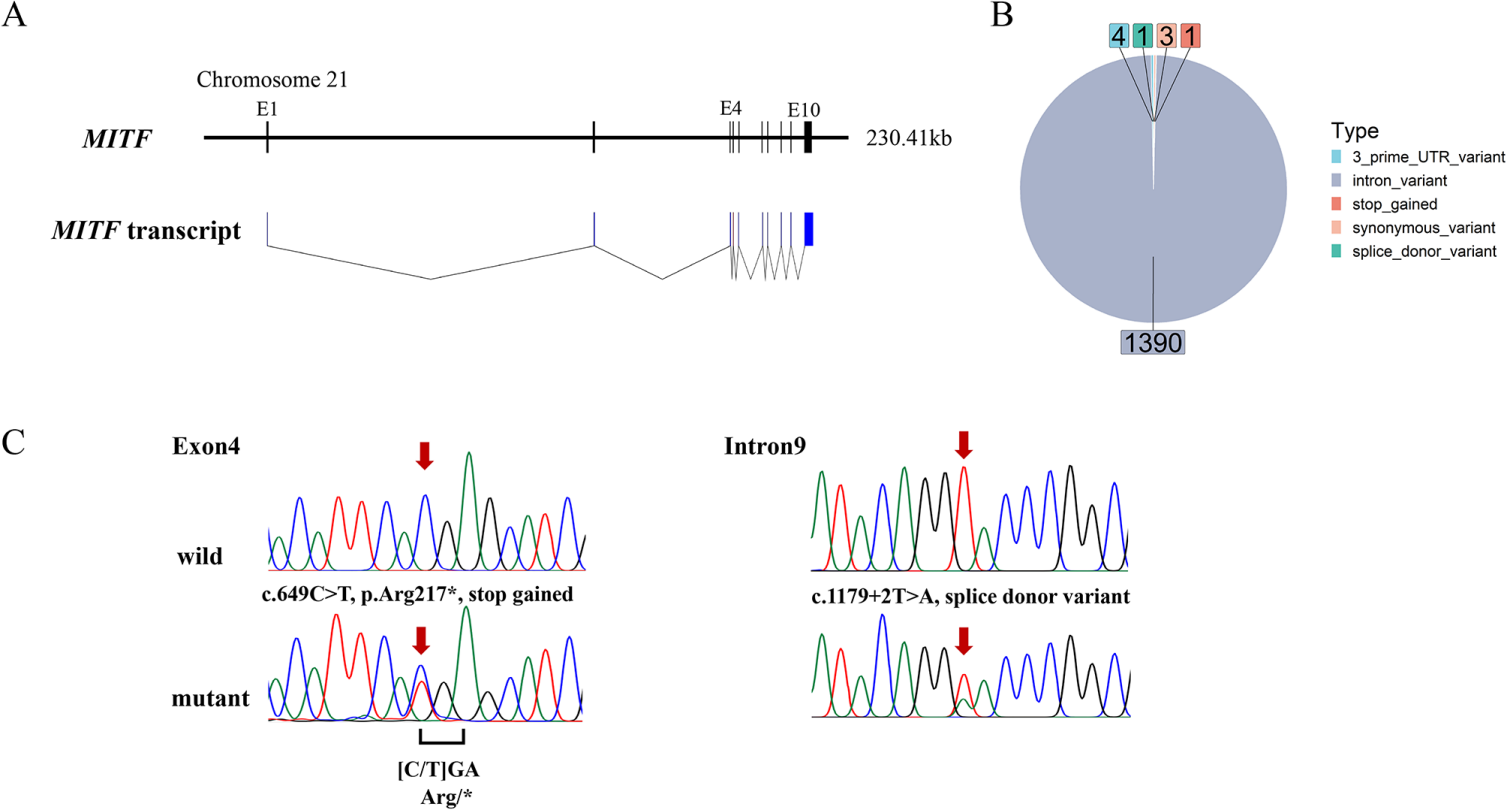
Identification of the nonsense and splice donor mutations in the *MITF* of the spotted buffaloes. **A***MITF* gene and transcript structure. **B** Types of variants annotated in *MITF*. **C** The Sanger sequencing of gDNA in solid and spotted buffaloes reveals a nonsense mutation in exon 4 and a splice donor mutation in intron 9 of *MITF*

The buffalo *MITF* gene spanned approximately 230 kb and comprised 10 coding exons (Fig. 1A, Figure S4A). A total of 1,399 SNPs were detected in the *MITF* genomic region, including 1,390 intron variants, three synonymous variants, four 3′ untranslated region (UTR) variants, one splice donor variant, and one nonsense variant (Fig. 1B).

The nonsense mutation (BBU21:31637770, c.649 C > T, Fig. 1C) led to a premature termination codon (PTC) (p.Arg217*) in the *MITF* exon 4. This variant is significantly association with the white-spotted phenotype (*P* = 3.45 × 10^− 8^, Table 1) and is also in linkage disequilibrium (LD) with the two adjacent variants (Figure S4B). The haplotype containing the nonsense mutation occurred exclusively in Indonesian swamp buffaloes and is one of the major haplotypes in spotted buffaloes (Figure S4C, D).

In addition, a splice donor variant (BBU21:31613452, c.1179 + 2T > A, Fig. 1C) was identified in intron 9 of the *MITF* gene. This variant was predicted to impact splicing with a SpliceAI donor loss delta score of 0.99 (Table 1). Again, this mutation is only present in Indonesian spotted buffaloes. Although this mutation with a non-reference allele frequency of 0.03 is not significantly associated with the white spotting (*P* = 0.11, Table 1), it was only found in one spotted buffalo without the *MITF* or *KIT* mutations and in three complete white buffaloes that were heterozygous for the *MITF* nonsense mutation. This may suggest that it is an alternative causative mutation for the white spotting and, together with a heterozygous nonsense genotype, for the complete white coat color. This observation supports a previous study in which the splice donor variant of *MITF* was found to be significantly associated with spotted color in swamp buffalo [19].

### Identification of nonsense mutation in *KIT* associated with the white spotting

Given the benefit of WGS data in exploring the contribution of rare variants to phenotypic variation [29, 30], we utilized the candidate gene approach to identify other functional mutations that may affect the white spotting.

Based on previous studies, we identified 11 plausible genes (see Methods). The interaction among these genes, as well as *MITF*, suggested their association with the white spotting (Table S3, Figure S5). Overall, 5,638 SNPs were found in the genomic regions of these 11 genes (Table S4). Functional annotations revealed 37 potential mutations affecting gene coding regions, including one nonsense mutation, 27 missense mutations, and nine splice region mutations. Based on the functional annotation and association analysis, these variants were further filtered as shown in Table 1.

In addition to the nonsense mutation in *KIT*, which was very likely to have a high impact, only two mutations in *ADAMTS20* and one in *TRPM1* were predicted to be deleterious. In addition, splice region mutations in *EDN3* and *PAX3* were predicted to have a low impact with a new splice donor site in *EDN3*. We further analyzed the association of these mutations with the white spotting and found only *KIT* and *PAX3* to be significantly associated.

**Table 1:** Functional mutations in potential candidate genes of the white spotting in swamp buffaloes

Gene	Ref/Alt	Position Chr.:Nucleotide	Alternative allele frequency	Type	*P*-value^a^	Impact^b^	Qualitative prediction/ Delta score (donor loss)^c^
*MITF*	C/T	21:31637770	0.28	stop gained	3.45 × 10^− 8^	high	-
*MITF*	T/A	21:31613452	0.03	splice donor	0.11	high	0.99
*KIT*	T/A	7:47207784	0.06	stop gained	0.01	high	-
*EDN3*	T/C	14:26511134	0.98	splice region	0.19	low	0.52
*PAX3*	C/T	2:163527488	0.27	splice region	0.013	low	0.00
*ADAMTS20*	G/C	4:83255631	0.06	missense	0.26	moderate	Deleterious, Probably damaging
*ADAMTS20*	A/C	4:83271344	0.25	missense	0.39	moderate	Deleterious, Probably damaging
*TRPM1*	G/A	20:42058056	0.37	missense	0.44	moderate	Deleterious (low confidence), Probably damaging

^a^The Fisher’s exact test (one-tailed) comparing the allele frequencies in solid and spotted buffaloes

^b^The effects of mutations predicted by the SnpEff

^c^Qualitative prediction of missense mutations using the SIFT 4G and PolyPhen-2, and effect prediction of splice site mutations using the SpliceAI

**Fig. 2:**
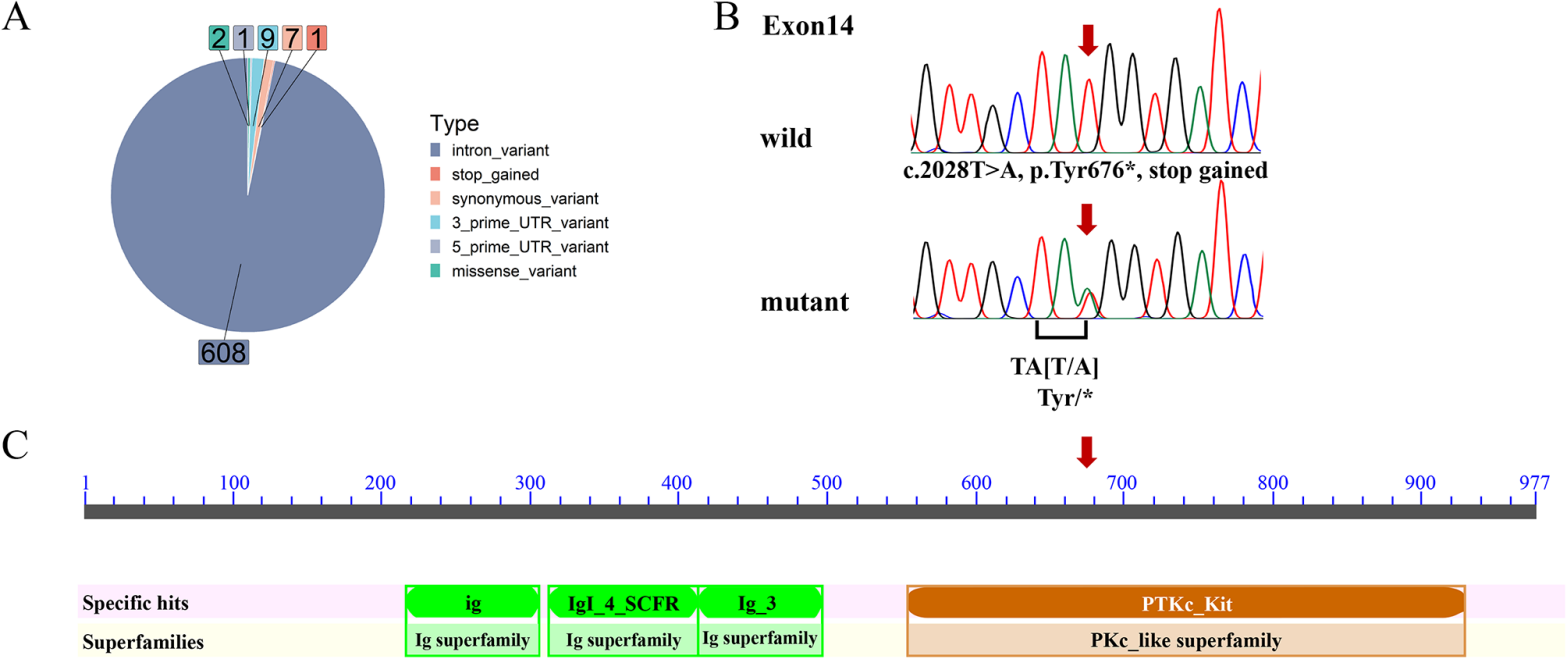
Identification of the nonsense mutation in the *KIT* of the spotted buffaloes. **A** Types of variants annotated in *KIT*. **B** The Sanger sequencing of gDNA in solid and spotted buffaloes reveals a nonsense mutation in exon 14 of *KIT*. **C** The domain structure of KIT

*KIT* encoded a tyrosine kinase receptor indispensable for the migration and survival of melanoblasts [31]. The buffalo *KIT* gene extended across 85.8 kb, encompassing 21 coding exons. Within the *KIT* genomic region, 628 SNPs were discovered, including 608 intron variants, seven synonymous variants, one 5′ UTR variant, nine 3′ UTR variants, two missense variants, and one nonsense variant (Fig. 2A). The nonsense mutation (BBU7:47207784, c.2028T > A, Fig. 2B) was located in its exon 14, altering the codon for tyrosine into a stop codon (p.Tyr676*), which truncated the tyrosine kinase domain (Fig. 2C). This mutation occurred exclusively in Indonesian spotted buffaloes and the genomic region encompassing the nonsense mutation was highly conserved. It was significantly associated with the white spotting (*P* = 0.01, Table 1).

The splice region mutation (BBU2:163527488, c.452–3 C > T) in *PAX3* was significantly associated with the white spotting (*P* = 0.013, Table 1). Although it was predicted to have a small effect that did not change protein structure (Table 1), it may be associated with a regulatory mutation to affect gene expression.

### *MITF* and *KIT* jointly explained most spotted phenotypes

Since both *MITF* and *KIT* were significantly associated with the white spotting, the genotype combinations of three candidate mutations were examined for their distribution in solid black and spotted buffaloes. Due to the lack of coverage at specific loci and the unavailability of DNA from one sample, we finally used 66 buffaloes for this analysis. Among the 39 spotted individuals, 37 carried one or more candidate mutations, while the remaining two were homozygous for the wild-type alleles (Table 2). Remarkably, the mutations in *MITF* and *KIT* jointly explained 95% of the white spotting in swamp buffaloes.

To further identify potential mutations affecting the remaining two spotted buffaloes, we filtered all mutations in the candidate genes, except *MITF* and *KIT*, and found 39 mutations in *SOX10*, *ADAMTS20*, *EDNRB*, and *TWIST2* to potentially contribute to the white spotting (Table S5). Of these mutations, four were significantly associated with the white spotting, but these were located in introns of *ADAMTS20* and *TWIST2* (*P* < 0.05, Table S6). One of these two spotted individuals was homozygous for mutations in *ADAMTS20* (BBU4:83215693, c.4294-1882T > A, Table 2) and *TWIST2* (BBU6:117577951, c.*35 + 19893G > A, Table 2), while another was homozygous for two mutations in *TWIST2* (BBU6:117578304, c.*35 + 20246G > A; BBU6:117580907, c.*35 + 22849G > A; Table 2). The *ADAMTS20* mutation also occurred in two solid black buffaloes (Table 2) and was therefore not causative for the coat color. It is noteworthy that the frequent heterozygotes of *MITF* 21:31637770 was only observed in white-spotted buffaloes if accompanied by a *KIT* mutation which also occurred in white-spotted animals together with the *MITF* homozygous wild-type genotype or one of the three *TWIST2* mutations (Table 2), supporting a potential causative role of these *TWIST2* mutations. Although our data indicated the involvement of other genes than *MITF* and *KIT* in the white-spotted trait, further investigation is warranted to identify different combinations of genotypes responsible for the white-spotted coat color in extended samples.

However, the analysis of WGS data often excludes intron variants or classifies them as variants of uncertain significance due to difficulties in predicting or determining their impacts [32]. Given the additional challenges in interpreting deep intronic mutations, the integration of multi-omics datasets will be critical to confirm their functions.

**Table 2:** The genotype combinations of eight candidate mutations in solid black, spotted, and pure white buffaloes

	Genotype combination								Phenotype				
*MITF* 21:31637770 C > T	*MITF* 21:31613452 T > A	*KIT* 7:47207784 T > A	*PAX3* 2:163527488 C > T	*ADAMTS20* 4:83215693 T > A	*TWIST2* 6:117577951 G > A	*TWIST2* 6:117578304 G > A	*TWIST2* 6:117580907 G > A		Solid black		White spotted		Pure white
**TT**	TT	TT	**TT**	TT	GG	GG	GG						1
**CT**	**TA**	TT	**CT**	TT	GG	GG	GG						1
**CT**	**TA**	TT	CC	TT	GG	GG	GG						2
**CT**	TT	**TA**	**TT**	TT	GG	**GA**	**GA**				2		
**CT**	TT	**TA**	**TT**	TT	GG	GG	GG				1		
**CT**	TT	**TA**	**CT**	TT	GG	GG	GG				1		
**CT**	TT	**TA**	CC	TT	GG	GG	GG				2		
**CT**	TT	TT	**CT**	TT	GG	GG	GG				13		
**CT**	TT	TT	CC	**TA**	**GA**	GG	GG				1		
**CT**	TT	TT	CC	**TA**	GG	GG	GG		1				
**CT**	TT	TT	CC	TT	GG	**GA**	**GA**				1		
**CT**	TT	TT	CC	TT	GG	**GA**	GG				1		
**CT**	TT	TT	CC	TT	GG	GG	GG		1		8		
CC	**TA**	TT	CC	TT	GG	GG	GG				1		
CC	TT	**TA**	**TT**	TT	**GA**	GG	GG				1		
CC	TT	**TA**	**TT**	TT	GG	GG	GG				1		
CC	TT	TT	**CT**	**TA**	GG	GG	GG		1				
CC	TT	TT	**CT**	TT	GG	**GA**	**GA**		1				
CC	TT	TT	**CT**	TT	GG	GG	GG		7				
CC	TT	TT	CC	**AA**	**AA**	GG	GG				1		
CC	TT	TT	CC	TT	GG	**AA**	**AA**				1		
CC	TT	TT	CC	TT	GG	**GA**	**GA**		3				
CC	TT	TT	CC	TT	GG	GG	GG		13				
						**Total**			**27**	**35**		**4**	

## Discussion

The white spotting, as a unique coat color pattern, has been mainly investigated in horses [11], cattle [8, 9], sheep [13, 14], and mice [16]. In this study, we integrated GWAS, selection signature analysis, and candidate gene approach to explore the molecular mechanism of the white spotting in swamp buffaloes. Previous study demonstrated that the two *MITF* mutations cannot fully account for the white spotting [19]. We successfully validated these two previously identified mutations in *MITF* [19] and detected the *KIT* gene as another candidate responsible for the manifestation of the white spotting in swamp buffaloes.

To date, a number of variants in *MITF* and *KIT* have been shown to cause the white spotting in domestic animals such as cattle [8, 9, 33, 34] and horses [7, 11, 35–39]. MITF is a transcription factor that has a bHLH-Zip domain, which not only influences melanocyte development, but also regulates the expression of color genes, including *TYR* and *TYRP1* [40]. Interestingly, a previous study of swamp buffalo *MITF* has demonstrated that the nonsense mutation resulted in mRNA degradation by the nonsense-mediated mRNA decay (NMD) pathway, while the splice donor mutation affected the binding efficiency of the mutant protein to the *MITF* binding site [19]. KIT, a type III receptor protein tyrosine kinase, plays a crucial role in the migration, survival, and proliferation of melanocytes and in the cell survival signaling of melanoblasts [31, 41–43]. Insufficient KIT expression in melanoblasts and melanocytes triggers apoptosis and results in the white spotting [38, 44]. Therefore, the nonsense mutation identified in the buffalo *KIT* gene in this study truncates the tyrosine kinase domain of KIT via the NMD pathway, which impacts the MAPK signaling pathway and thereby regulates melanocyte development. Most spotted buffaloes in this study can be classified mainly into three categories: those with *MITF* or *KIT* mutation or with both *MITF* and *KIT* mutations. These mutations might lead to reduction in MITF or KIT protein levels, which impair melanocyte development and cause the white spotted coat color.

The white phenotype in mammals can also be caused by the disruption of melanin synthesis [38, 45, 46]. The causal mutation for dominant white coat color in swamp buffaloes from China is a LINE-1 insertion in the *ASIP* gene [47]. To determine whether the four completely white Indonesian buffaloes in our study were caused by the *ASIP* mutation, we investigated their whole genome sequencing reads, but did not find such LINE-1 insertion (Figure S6). *ASIP* and *MITF* play different roles in the formation of white coat color. Specifically, over-expression of *ASIP* prevents melanocytes from undergoing terminal differentiation [47, 48], while *MITF* participates in melanocyte development by modulating the expression of target genes [49–52]. In mice, early deletion of functional MITF protein leads to the termination of melanocyte development [53], with individuals harboring a homozygous mutation presenting with a white coat and small eyes [54, 55]. In our study, buffaloes homozygous for the nonsense mutation in *MITF* are viable and typically have a more pronounced depigmentation phenotype than heterozygous buffaloes. One of the four white individuals was homozygous for the *MITF* nonsense mutation, while the remaining three were carriers of the nonsense mutation and splice donor mutation in *MITF*, which most likely resulted in an abnormal MITF protein through NMD and abnormal splicing. Therefore, we propose that the white buffaloes in our study lacked normal MITF protein and thus their melanocyte development was disrupted.

A previous study reported a significant association between the splice donor mutation of *MITF* and white-spotted coat color in Indonesian swamp buffalo [19]. This study, however, detected only a limited number of individuals carrying this mutation and reported a non-significant association with white spotting phenotype. In addition, the candidate mutations in *ADAMTS20* and *TWIST2* were found at a low frequency. Therefore, these candidate functional variants require the validation on the basis of a larger panel of spotted and white swamp buffaloes.

## Conclusions

Our results first emphasized previous findings that suggested the involvement of the *MITF* gene in the white spotted phenotype in water buffaloes and then revealed a new mutation in the *KIT* gene. These mutations jointly explained 95% of the white spotting in swamp buffaloes and may regulate melanocyte development by impacting gene function, ultimately resulting in the white spotting. We also found evidence for other candidate genes relevant to the white spotting. The remarkable diversity of spotted-associated genotypes may reflect a strong selection of *de novo* mutations that confer the highly prized spotted phenotype. However, genomic analysis of more white-spotted buffaloes is necessary to confirm and/or identify other causal variants that could fully explain the four different spotting patterns according to the Toraja classification system.

## Methods

### Samples included in this study

A total of 67 swamp buffaloes were sampled from local village farms on Sulawesi and Sumbawa islands (Figure S7A) in Indonesia, consisting of 28 solid black (wild type), 35 spotted, and four pure white individuals (Table S7). Hair follicle samples were collected from the tail. Pedigree information was available for 16 of these individuals (Table S8). White spotting was scored as the presence or absence of white spots on the coat. The complete whiteness can be regarded as an extreme form of white spotting [56].

### Sequencing and variant calling

Genomic DNA was extracted from hair follicles using the phenol/chloroform method. The quality and quantity of the genomic DNA were assessed and verified using agarose gel electrophoresis and a Nanodrop spectrophotometer (Thermo Fisher Scientific, Waltham, MA, USA). Paired-end libraries were prepared with an average insert size of 350 bp and sequenced on the Illumina HiSeq X Ten Platform (Illumina, San Diego, CA, USA). Raw reads were filtered using the QualityTrim software (https://bitbucket.org/arobinson/qualitytrim). The reads were then aligned to the water buffalo reference genome (UOA_WB_1 at https://www.ncbi.nlm.nih.gov/datasets/genome/GCF_003121395.1/) [57] using the BWA-MEM algorithm [58]. The UOA_WB_1 is a chromosome level reference genome with N50 and L50 scores similar to those of a recently published water buffalo reference genome (https://www.ncbi.nlm.nih.gov/datasets/genome/GCF_019923935.1/). After sorting the mapping reads using Samtools [59], potential PCR duplicates were marked with the “MarkDuplicates” of Picard tools v2.9.0 (http://broadinstitute.github.io/picard) and local realignment around indels was performed by the Genome Analysis Toolkit (GATK, v3.7) [60]. We obtained sequence data with an average depth of 9.73× and a coverage of 98.27% (Table S7). SNPs were identified using the “UnifiedGenotyper” of GATK. Finally, hard filters were applied to the raw SNPs according to the following parameters: QUAL < 30; QualByDepth (QD) < 2.0; RMSMappingQuality (MQ) < 40.0; MappingQualityRankSumTest (MQRankSum) < − 12.5; ReadPosRankSumTest (ReadPosRankSum) < − 8.0; and HaplotypeScore > 13.0.

## Quality control

The variant call format (VCF) file generated in GATK was converted to the Plink format using the PLINK software v1.90 [61]. All the variants were filtered by PLINK with the following options “--mind 0.1, --maf 0.05, --geno 0.1, --hwe 0.0000000001 in the cases and --hwe 0.000001 in the controls”. Using these parameters, we excluded samples with missing call rates exceeding 0.1 and removed SNPs with minor allele frequencies (MAF) below 0.05, SNP missing call rates exceeding 0.1, and Hardy-Weinberg equilibrium (HWE) exact test p-values below 10^− 10^ in cases and below 10^− 6^ in controls. Autosomal SNPs were pruned using the PLINK software v1.90 [61], with a window size of 50 SNPs, a step of 5 SNPs, and*r*^*2*^ threshold of 0.1, resulting in 388,771 independent SNPs for calculating the GWAS threshold.

### Genome-wide association study

A principal component analysis (PCA) revealed no clear distinct differentiation between the spotted and solid black buffaloes (Figure S7B), ensuring that the association test was not influenced by potential population stratification. The association test was performed with a univariate linear mixed model using the GEMMA software v0.98.3 [62] with statistical model y = Wα + xβ + u + ε, where y is a vector of phenotype values for 67 individuals, coded as 0 for solid and 1 for spotted; W is an n × c matrix of covariates (PC1, PC2); α is a c × 1 vector of the corresponding coefficients, including the intercept; x is an *n* × 1 vector of marker genotypes; β is the effect size of the marker; u is an *n* × 1 vector of random polygenic effects with a covariance structure as u ~ N (0, KVg), where K is an n × n marker-based additive genetic relatedness matrix and Vg is the polygenic additive variance; and ε is an *n* × 1 vector of residual errors with ε ~ N (0, IVe), where I is an n × n identity matrix and Ve is the residual variance.

The threshold *P*-value for suggestive association was 2.57 × 10^–6^ (1/388,771). The Manhattan and quantile–quantile (Q-Q) plots were created using the ggplot2 [63] and qqman [64] packages in R, respectively.

### Detection of selection signature

To identify potential selection signatures across the genome, XP-EHH was employed to identify differences between populations using Selscan v1.2.0a [65]. Haplotype phasing was first implemented in BEAGLE [66]. SNPs with minor allele count (MAC) lower than 10 and samples with a fraction of missing genotypes (F_MISSING) higher than 0.1 were removed using the bcftools v1.17 [59]. We identified regions under selection in the spotted buffaloes in contrast to the solid black buffaloes based on the extended haplotype statistics via the XP-EHH approach, using 50 kb sliding windows with a step size of 20 kb. For the XP-EHH selection scan, our test statistic was the average XP-EHH score in each 50 kb region. Positive or negative values of XP-EHH indicated selection in spotted or solid black buffaloes, respectively. The top 5% windows with average XP-EHH values were considered as the candidate selection regions and were annotated by the ANNOVAR [67]. The clusterProfiler R package [68] was used for the GO analysis. We selected GO terms with *P* < 0.05.

### Selection of candidate genes responsible for white-spotted coat color

Candidate genes for white spotting selected from published studies included *KIT* [33], *KITLG* [69], *EDN3* [15], *EDNRB* [16], *SNAI2* [17], *TRPM1* [70], *ADAMTS20* [18], *TWIST2* [10], and the *MITF* transcription factors *PAX3* [71], *SOX10* [72], and *LEF1* [28] (Table S9). A protein-protein interaction (PPI) network was predicted by the candidate gene products using the STRING database [73] and GeneMANIA web site [74]. The interactions were categorized into four groups in the STRING database on the basis of the combined score of each interaction: low, medium, high, and the highest confidences at 0.15, 0.4, 0.7, and 0.9, respectively.

### Transcript assembly

The RNA-seq data of skin tissues for three solid black and three white buffaloes [47] were collected to analyze the transcripts of candidate genes. Genome-guided transcript assembly was performed using the StringTie [75]. The RNA-seq reads were visualized in the Integrative Genomic Viewer (IGV) [76].

### Functional annotation and filtering of mutations

The SnpEff [77] was used to annotate and predict the variants’ effects in candidate genes with default parameters. Nonsense, missense, and splice site mutations were selected by the SnpSift [78] with the parameter “ANN[*].EFFECT”. The pathogenicity of missense mutations was analyzed using the SIFT 4G [79] and PolyPhen-2 [80]. The effects of variants on splicing were predicted using the SpliceAI and the resulting scores were evaluated as 0.2 for high recall, 0.5 for recommended, and 0.8 for high precision [81]. For the spotted buffaloes that did not carry the *MITF* and *KIT* candidate mutations, the vcfR R package [82] was used to analyze their genotype frequencies along with the solid black buffaloes at all candidate mutations, based on dominant and recessive inheritance patterns. The Fisher’s exact test (http://vassarstats.net/) was performed to verify the association between the allele frequency of SNPs and the coat color patterns (spotted and solid).

### Linkage disequilibrium and haplotype analyses

The Haploview software v4.2 [83] was used to analyze the LD and haplotypes of candidate genes. LD blocks were defined according to the definition of Gabriel et al. [84].

### Verification, frequency, and conservation of candidate mutations

PCR primers (Table S10) were designed by Primer3 v0.4.0 [85, 86]. PCR products amplified in a 25-µl reaction containing 50 ng genomic DNA, 21 µl Golden Mix (Tsingke, Beijing, China), and 10 pmol forward and reverse primers were used for the Sanger sequencing.

Genomic data from 45 buffalo populations (348 individuals, Table S11) were used to calculate the frequency of candidate SNPs by the PLINK v1.90 [61]. Multiple protein alignments were visualized and analyzed using the Unipro UGENE v38.1 [87].

### Electronic supplementary material

Below is the link to the electronic supplementary material.

Supplementary Material 1

Supplementary Material 2

## Data Availability

The newly generated genome sequences of Indonesian buffalo are available from the Sequence Read Archive (SRA) with the Bioproject accession number PRJNA1053598 and PRJNA1135737.

## Abbreviations

GWAS: Genome-Wide Association Study
WGS: Whole-Genome Sequencing
SNPs: Single Nucleotide Polymorphisms
PCA: Principal Component Analysis
VCF: Variant Call Format
GATK: Genome Analysis Toolkit
Q-Q: Quantile–Quantile
MAC: Minor Allele Count
F_MISSING: Fraction of Missing Genotypes
XP-EHH: Cross-population extended haplotype homozygosity
GO: Gene Ontology
PPI: Protein-Protein Interaction
LD: Linkage Disequilibrium
IGV: Integrative Genomic Viewer
UTR: Untranslated Region
PTC: Premature Termination Codon
NMD: Nonsense-mediated mRNA decay
