# Supplementary material for: Genomic analysis reveals the association of KIT and MITF variants with the white spotting in swamp buffaloes
Source: BMC Genomics. 2024 Jul 24;25:713. doi: 10.1186/s12864-024-10634-2 (PMC11267946; doi:10.1186/s12864-024-10634-2)
Supplement: Supplementary file 2 — Supplementary Material 2 [file 12864_2024_10634_MOESM2_ESM.docx]

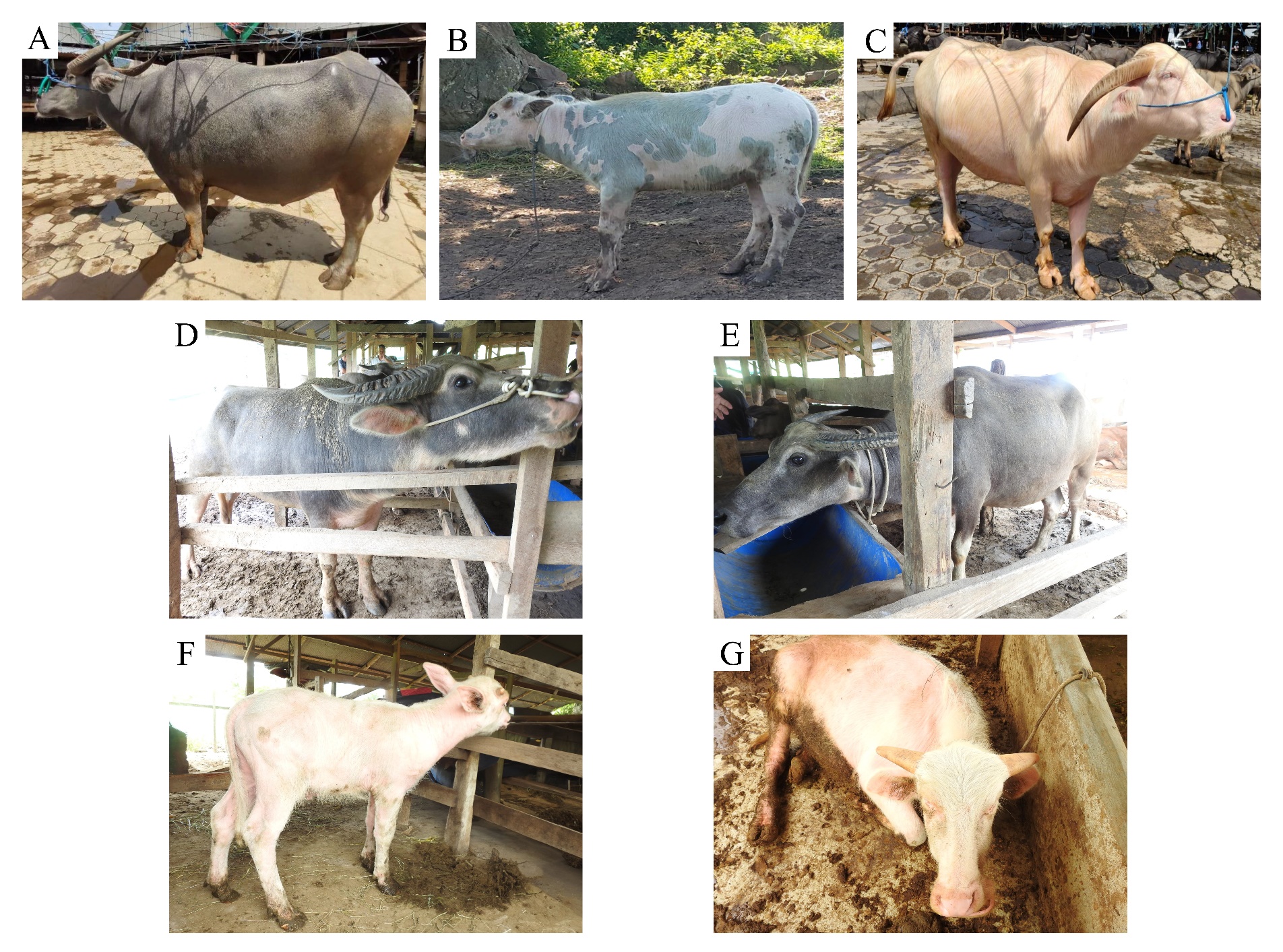


**Figure S1.** **Coat color phenotype of Indonesian buffaloes. A** solid black. **B** spotted. **C** pure white. **D** Male spotted (Father). **E** Female spotted (Mother). **F** Male calf (pure white, calf). **G** Female calf (pure white, calf).


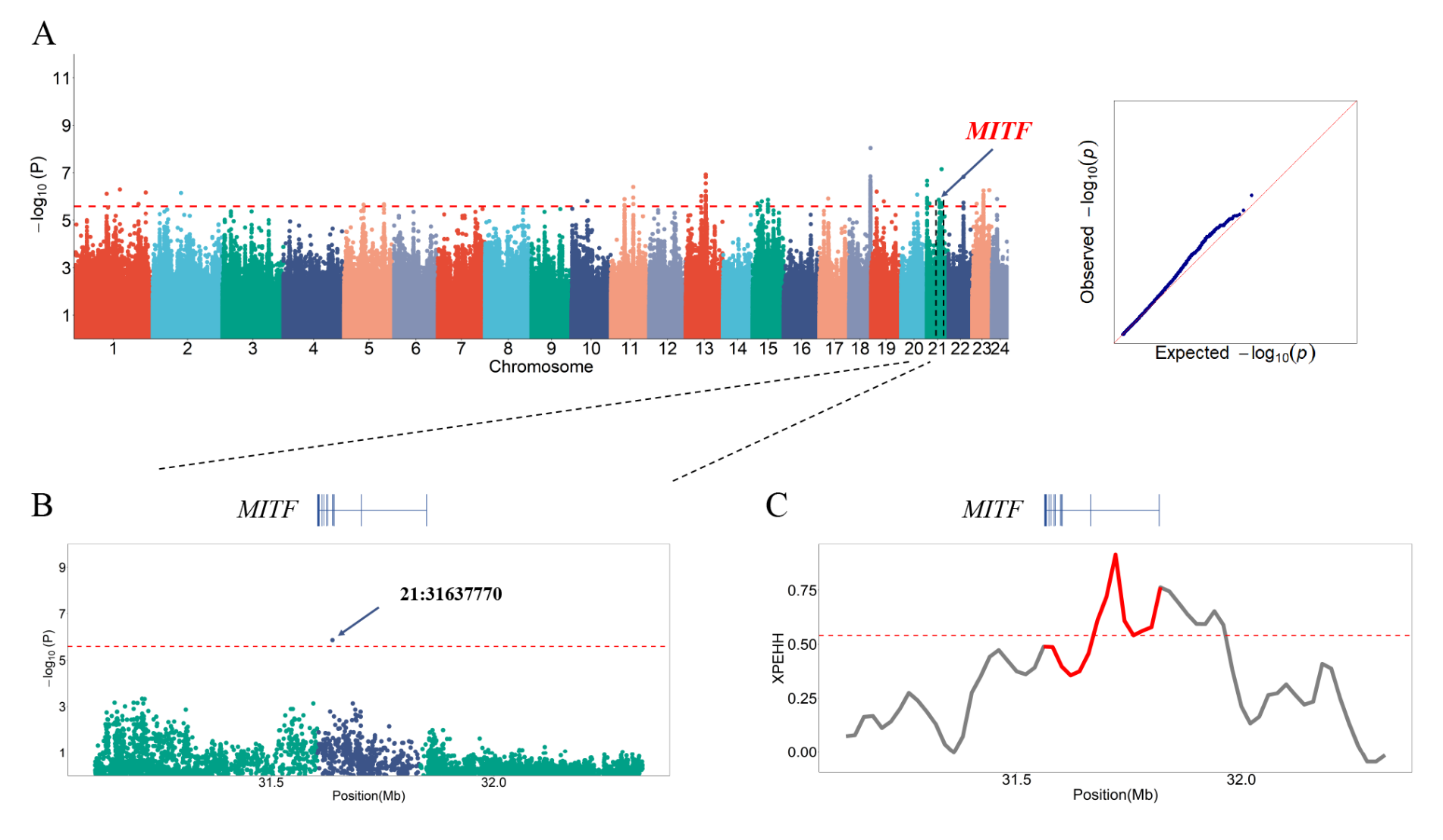


**Figure S2.** **Genomic analyses identified *MITF* on buffalo chromosome 21 as a potential candidate gene for white spotting. A** The Manhattan and Q-Q plot of GWAS. Red line indicates the suggestive significance (2.57 × 10^–6^) threshold. The QQ plot shows the deviation of observed p-values from expected p-values. **B** Zoomed association signals of GWAS in the *MITF* genomic region (including 0.5 Mb each up and down-stream of the *MITF* gene). **C** Zoomed association signals of XP-EHH in the *MITF* genomic region (including 0.5 Mb each up and down-stream of the *MITF* gene).


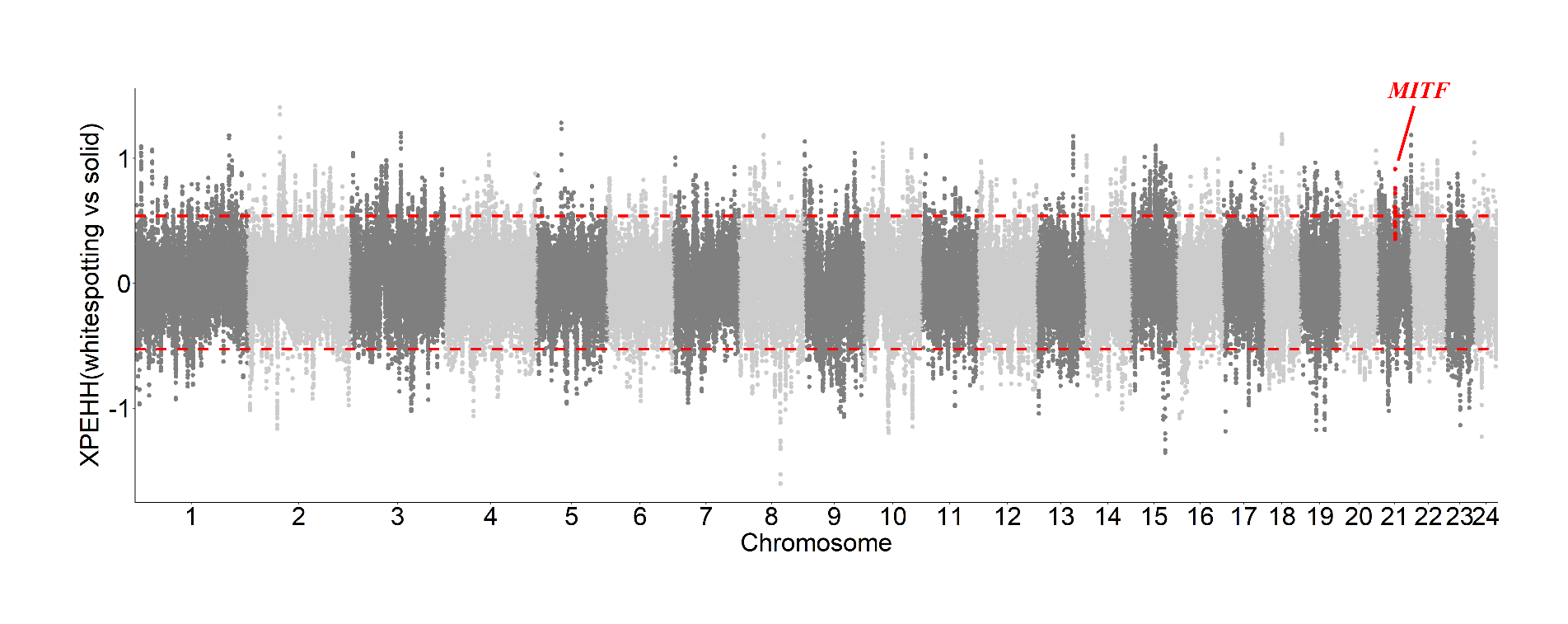


**Figure S3. Selection signature analysis using the XP-EHH method.** The manhattan plot reflects the distribution of XP-EHH score. The solid buffaloes are defined as reference population and the spotted buffaloes are defined as observed population. Red line indicates the top 5% suggestive line.


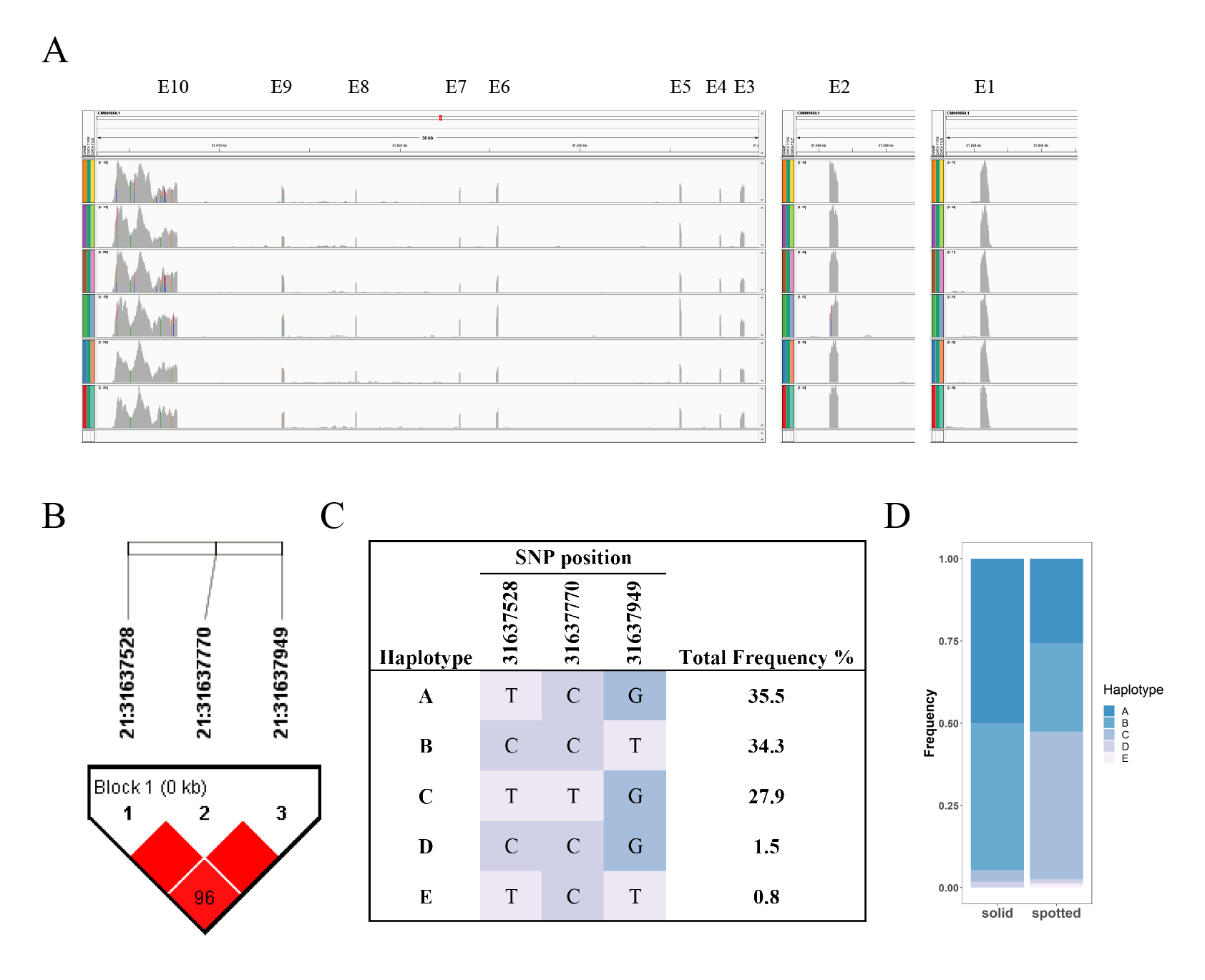


**Figure S4.** **The *MITF* transcript and the haplotype containing the *MITF* nonsense mutation. A** IGV illustration of the mapped RNA-seq reads in *MITF*. **B** Linkage disequilibrium (LD) among the three SNPs of the *MITF* gene. **C** The LD block defines five haplotypes within the sampled buffalo population. D The frequency of the haplotypes in solid and spotted buffaloes.


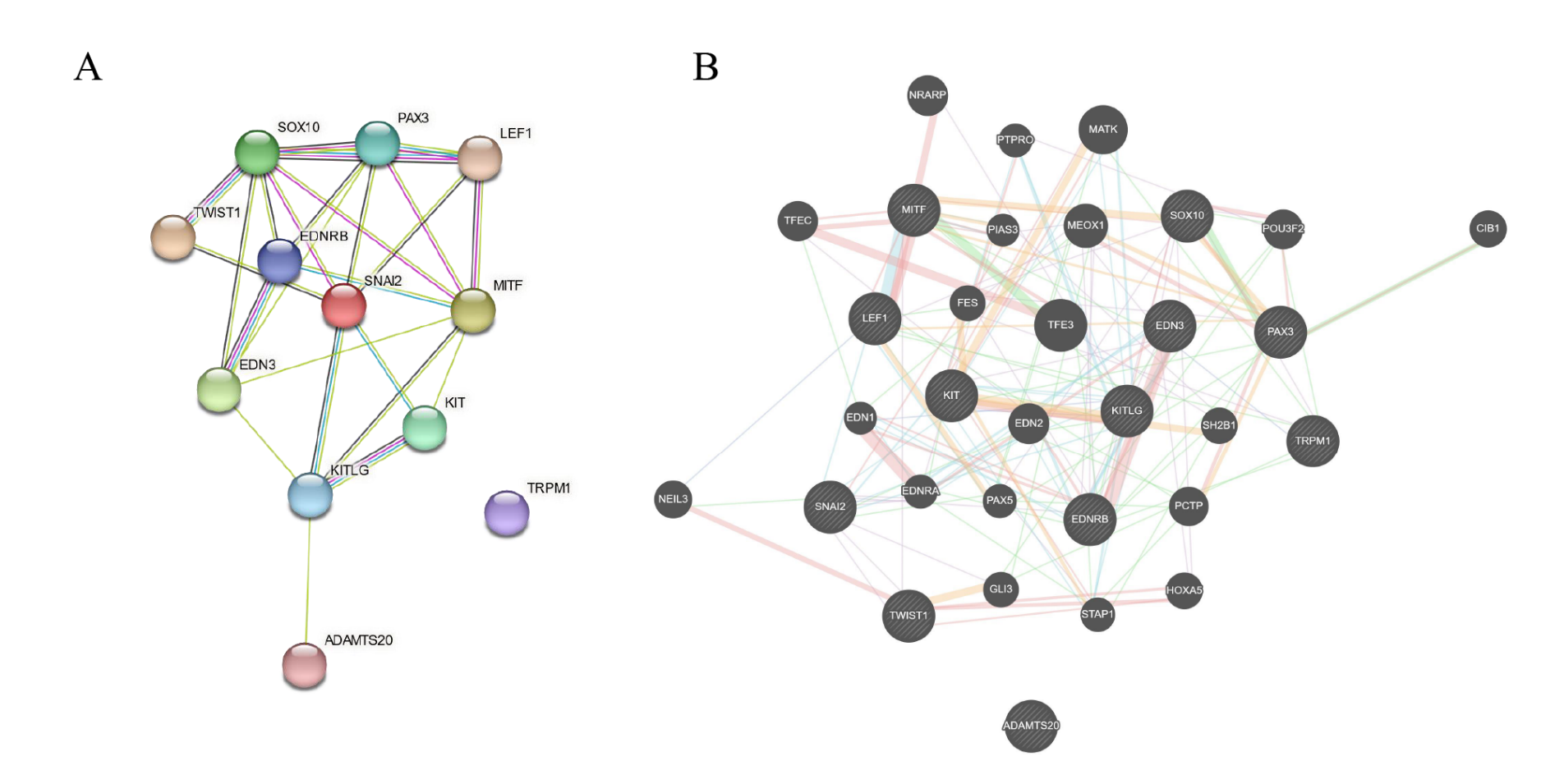


**Figure S5. The protein–protein interaction (PPI) network of selected genes associated with the white spotting. A** PPI network analysis using STRING database. **B** PPI network constructed with GeneMANIA.


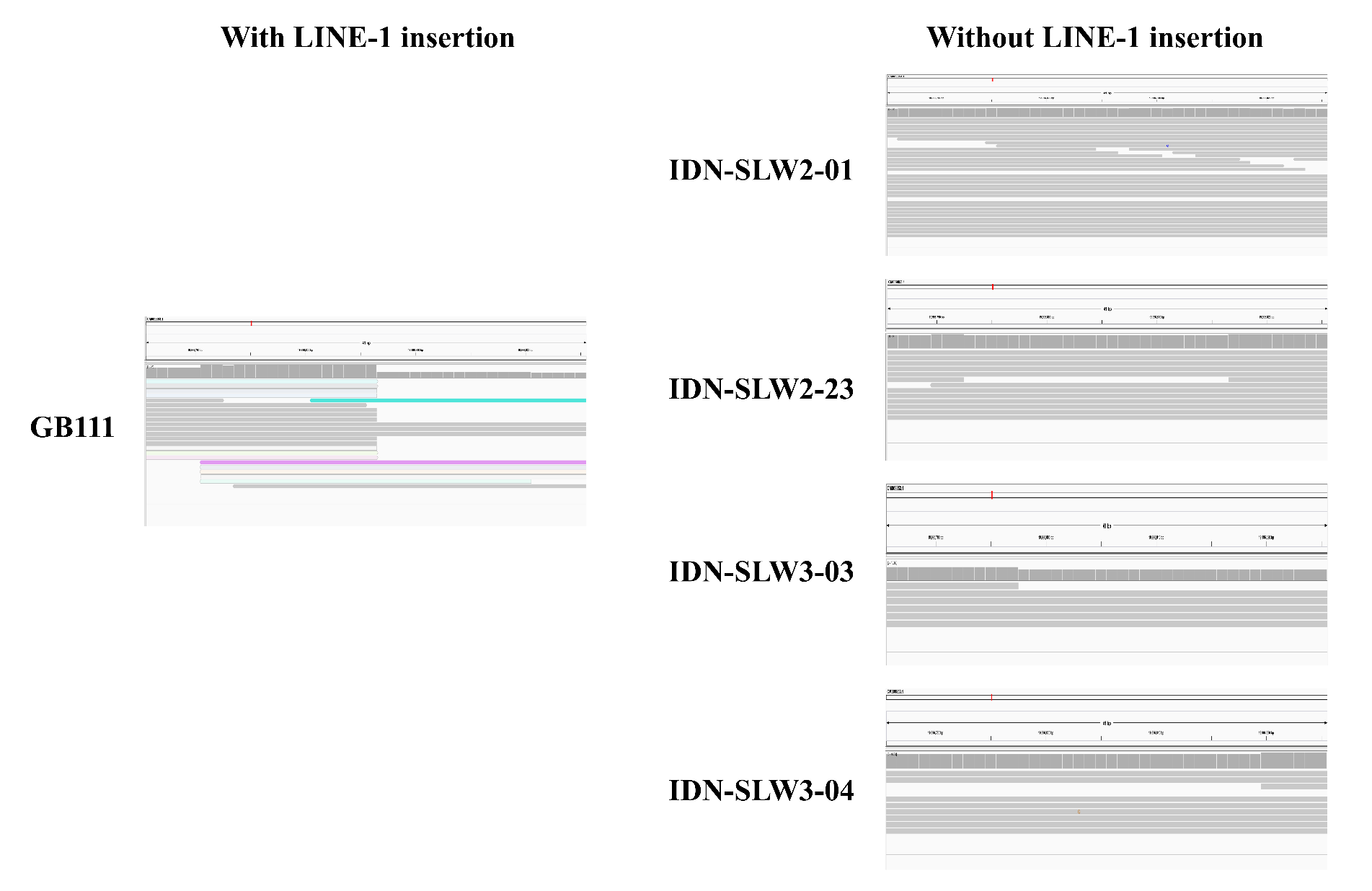


**Figure S6. The visualization results at BBU14:19996806 using the Integrative Genomic Viewer (IGV).** The left column represents an individual with LINE-1 insertion and the right represents the four individuals without LINE-1 insertion in this study.


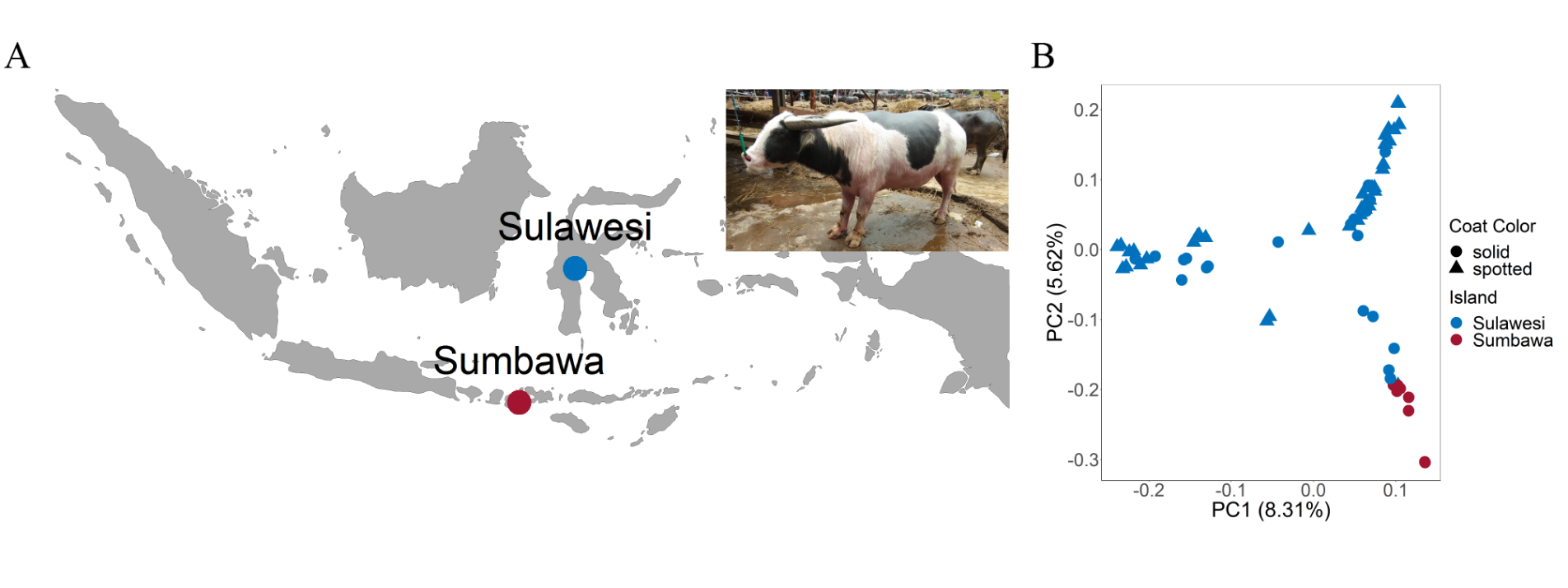


**Figure S7.** **Geographical location of the samples and principal component analysis. A** The buffaloes in this study were sampled from Sulawesi and Sumbawa islands in Indonesia. **B** The principal component analysis of whole genome sequence data was performed using the GCTA v1.93.3 beta (https://github.com/jianyangqt/gcta).
